# Arecoline induced disruption of expression and localization of the tight junctional protein ZO-1 is dependent on the HER 2 expression in human endometrial Ishikawa cells

**DOI:** 10.1186/1471-2121-11-53

**Published:** 2010-07-06

**Authors:** Sarbani Giri, Kevin M Poindexter, Shyam N Sundar, Gary L Firestone

**Affiliations:** 1 Department of Molecular and Cell Biology, University of California, Berkeley, CA, 94720, USA; 2 Department of Life Science, Assam University, Silchar, Assam-788011, India

## Abstract

**Background:**

Approximately 600 million people chew Betel nut, making this practice the fourth most popular oral habit in the world. Arecoline, the major alkaloid present in betel nut is one of the causative agents for precancerous lesions and several cancers of mouth among those who chew betel nut. Arecoline can be detected in the human embryonic tissue and is correlated to low birth weight of newborns whose mothers chew betel nut during pregnancy, suggesting that arecoline can induce many systemic effects. However, few reports exist as to the effects of arecoline in human tissues other than oral cancer cell lines. Furthermore, in any system, virtually nothing is known about the cellular effects of arecoline treatment on membrane associated signaling components of human cancer cells.

**Results:**

Using the human Ishikawa endometrial cancer cell line, we investigated the effects of arecoline on expression, localization and functional connections between the ZO-1 tight junction protein and the HER2 EGF receptor family member. Treatment of Ishikawa cells with arecoline coordinately down-regulated expression of both ZO-1 and HER2 protein and transcripts in a dose dependent manner. Biochemical fractionation of cells as well as indirect immunofluorescence revealed that arecoline disrupted the localization of ZO-1 to the junctional complex at the cell periphery. Compared to control transfected cells, ectopic expression of exogenous HER2 prevented the arecoline mediated down-regulation of ZO-1 expression and restored the localization of ZO-1 to the cell periphery. Furthermore, treatment with dexamethasone, a synthetic glucocorticoid reported to up-regulate expression of HER2 in Ishikawa cells, precluded arecoline from down-regulating ZO-1 expression and disrupting ZO-1 localization.

**Conclusion:**

Arecoline is known to induce precancerous lesions and cancer in the oral cavity of betel nut users. The arecoline down-regulation of ZO-1 expression and subcellular distribution suggests that arecoline potentially disrupts cell-cell interactions mediated by ZO-1, which may play a role in arecoline-mediated carcinogenesis. Furthermore, our study has uncovered the dependency of ZO-1 localization and expression on HER2 expression, which has therefore established a new cellular link between HER2 mediated signaling and apical junction formation involving ZO-1.

## Background

Areca nut (*Areca catechu *Linn) chewing in the form of betel quid is popular in southeast Asian countries and plays a major role in the pathogenesis of precancerous lesions and several cancer of the oral cavity, including precancerous lesions such as leukoplakia and oral submucous fibrosis [1,2]. Epidemiological studies also indicate adverse birth outcome including spontaneous abortion, still birth, low birth weight and birth length reduction among pregnant women who consumed betel quid during pregnancy [3,4]. The meconium, urine and cord serum of newborns whose mother chewed betelquid during pregnancy was found to contain arecoline as detected by mass spectrometric assays[5]. Arecoline and its derivatives are being used clinically to treat Alzheimer's disease based on their use as centrally active muscarinic agents [6].

The mechanism of arecoline mediated carcinogenesis in the oral cavity is not fully understood. However, there are reports which indicate that arecoline induces immunodepression, hepatotoxicity and depression of natural antioxidants such as superoxide dismutase, catalase, reduced glutathione and glutathione-s-transferase that are known to neutralize reactive oxygen species in mice [7]. Arecoline has also been found to elicit mutagenicity, genotoxicity, cytotoxicity and chromosomal aberration in different biological systems [8], and has been shown to mediate the cell cycle arrest, ROS generation, change in the mitochondrial membrane potentials in oral mucosal fibroblasts and oral KB epithelial cells [9]. Furthermore, arecoline was recently reported to alter metallothionein-1 [10] and Heme Oxygenase-1 expression [11,12] in clinicopathological profile of oral submucous fibrosis samples. Our earlier study shows that arecoline is metabolized to N-oxide of arecoline in mouse *in vivo *and human *in vitro*, which is Flavin monooxygenase-1 dependent [13,14]. Thus, exposure to arecoline has pleiotropic responses in a variety of tissue types that together account for its carcinogenic properties.

Relatively little is known about the potential cellular effects of arecoline on plasma membrane associated signaling components in human cancers. Two types of plasma membrane signaling components that can be altered in transformed cells are apical junction proteins involved in regulating cell-cell interactions and members of specific tyrosine kinase receptors. Tight junctions comprise the more apical structure of junctional complexes that restrict solute diffusion along the paracellular space conferring barrier properties to epithelial and endothelial sheets. Loss of normal junctional formation and cell-cell interactions is thought to play an important role in cancer progression due to significant changes in epithelial compartmentalization and the tissue microenvironment. A key component of junctional complexes that regulates tight junction formation is zonula occludens-1 (ZO-1) [15]. Z0-1 is a 220 kDa protein member of the MAGUK (membrane-associated guanylate kinase homologs) gene family that interacts directly with the transmembrane protein occludin, with ZO-2 and with AF- 6, a target of the *ras *oncogene, which is involved in acute myeloid leukemia [16]. ZO-1 is an important marker for tight junction integrity, which is disrupted in many intestinal diseases and highly invasive cancer types, and has been shown to be down regulated in poorly differentiated, highly invasive breast cancer cell lines [17]. Immunohistochemical analysis revealed a gradual decrease of ZO-1 protein from normal breast tissue to well differentiate to moderately differentiate to poorly differentiate human breast cancer tissue samples [18].

HER2 is a transmembrane tyrosine kinase receptor that is a member of the epidermal growth factor (EGF) receptor gene family [19,20] that is expressed at high levels in several human cancers including in late stage endometrial carcinomas and other reproductive cancers [20-22]. Expression of the HER2 gene has been extensively studied in a variety of ovarian and breast adenocarcinomas, with most studies correlating HER2 overexpression with a poor prognosis. Steroid hormones can alter the expression of HER2 in these two types of tumors. For example, in human neoplastic mammary cells estrogens inhibit HER2 expression [23], whereas, in ovarian adenocarcinoma cells glucocorticoids exert a stabilizing effect on existing HER2 transcripts [24].

In the present study, we have established in human Ishikawa endometrial cancer cells that arecoline downregulates expression and disrupts the junctional localization of ZO-1 in a process that requires the downregulation of HER2. Our findings implicate a role for HER2 signaling in the arecoline disruption of apical junction organization in human cancer cells, and have uncovered a new cellular link between HER2 and the control of ZO-1 expression and localization.

## Methods

Dulbecco's modified Eagle's medium, fetal bovine serum (FBS), calcium- and magnesium-free phosphate-buffered saline, L-glutamine and trypsin-versene mixtures were purchased from Biowhittaker (Walkersville, MD). Insulin (bovine) and dimethyl sulfoxide (DMSO) were purchased from Sigma Chemical Co. (St Louis, MO). Arecoline hydrobromide was purchased from Aldrich (Milwaukee, WI). The sources of other reagents are either listed below were of the highest purity available. All antibodies were purchased from Santa Cruz Biotechnology (Santa Cruz, CA) and Invitrogen.MG132 and Dexamethasone were purchased from Sigma Chemical Co.

### Cell culture

Ishikawa human endometrial adenocarcinoma cells were obtained from American Type Culture Collection (Manassas, VA). Ishikawa cells were grown in Dulbecco's modified Eagle's medium supplemented with 10% Fetal bovine Serum, 10 μg/ml bovine insulin and 50 U/ml penicillin, 50 U/ml streptomycin and 2 mM Lglutamine. The cells were grown to subconfluency in a humidified air chamber at 37°C containing 5% CO2. Arecoline (99.9% high-performance liquid chromatography grade) was dissolved in appropriate concentrations in DMSO. DMSO was used as vehicle control for all experiments. All the experiments utilized cultured Ishikawa cells in passage 25 to passage 28.

### Western Blot Analysis

After the indicated treatments, cells were harvested in radioimmune precipitation assay buffer (150 mM NaCl, 0.5% deoxycholate, 0.1% NoNidet-p40 (Nonidet P-40, Flulta Biochemitra, Switzerland), 0.1% SDS, 50 mM Tris) containing protease and phosphatase inhibitors (50 g/ml phenylmethylsulfonyl fluoride, 10 g/ml aprotinin, 5 g/ml leupeptin,0.1 g/ml NaF, 1 mM dithiothreitol, 0.1 mM sodium orthovanadate, and 0.1 mM_-glycerol phosphate). These extracts were then quantified using the Lowry Method (Bio-Rad Laboratories, Hercules, CA). Equal amounts of total cellular protein were mixed with loading buffer (25% glycerol, 0.075% SDS, 1.25 ml β-mercaptoethanol,10% bromphenol blue, 3.13% 0.5 M Tris-HCl, and 0.4% SDS (pH 6.8) and fractionated on 10% polyacrylamide/0.1% SDS resolving gels by electrophoresis. Spectra Multicolor Broad range Protein Ladder from Fermentas life sciences was used as the molecular weight standard. Proteins were electrically transferred to nitrocellulose membranes (Micron Separations, Inc., Westboro, MA). Equal protein loading was confirmed by Ponceau S staining of blotted membranes. Proteins were blocked for one and half hour at room temperature with Western wash buffer-5% NFDM (10 mM Tris-HCl (pH 8.0), 150 mM NaCl, and 0.05% Tween 20, 5% nonfat dry milk). Protein blots were subsequently incubated for overnight at 4 degree temperature with antibody in western buffer. The antibodies used were rabbit anti-ZO-1 (Invitrogen); rabbit anti-Claudin-1 (Santa Cruz Biotechnology); rabbit anti-E-cadherin (Santa Cruz Biotechnology); rabbit anti-beta-catenin (Santa Cruz Biotechnology); and rabbit anti-HER2/neu (Santa cruz Biotechnology). The working concentration for all antibodies was 1 μl/ml in Western wash buffer. Immunoreactive proteins were detected after incubation with horseradish peroxidase conjugated secondary antibody diluted to 0.25 μl/ml in Western wash buffer (goat anti-rabbit IgG and rabbit anti-mouse IgG (Bio-Rad). Blots were treated with ECL western blotting detection reagent (GE healthcare) and detected on the high performance chemiluminescence film (GE healthcare, UK).

### Reverse Transcription PCR

Ishikawa cells were harvested in PBS and total RNA was isolated. RNA was quantified. 5 μg of total RNA was subjected to reverse transcription using murine myelogenous leukemia reverse transcriptase with First strand Buffer, random Primer (hexamers), dNTPs. 2 μl of cDNA was then subjected to PCR using Platinum *Taq*, 10 × PCR buffer, and 200 μM each dNTP (Invitrogen) along with the following primer sets and conditions: HER2 Forward 5'-CCAGCTCTTTGAGGACAACT - 3' and Reverse 5'-ATGTCCTTCCACAAAATCGT- 3', and the cycling conditions were 30 seconds at 95°C followed by 30 seconds at 52°C for annealing and finally 30 seconds at 72°C for extension for 26 cycles. ZO-1 Forward 5'-CGAGTTGCAATGGTTAACGGA-3' and Reverse 5' -TCAGGATCAGGACGACTTACTGG- 3', and the cycling conditions were 30 seconds at 95°C followed by 30 seconds at 55°C for annealing and finally 30 seconds at 72°C for extension for 26 cycles. GAPDH primers 5'-TGAAGGTCGGAGTCAACGGATTTG-3', GAPDH Reverse: 5'-CATGTGGGCCATGAGGTCCACCAC-3' (Ambion, Austin TX) served as a control, and PCR was performed according to the manufacturer's instructions. The PCR products were run on 1.1% agarose gels with Ethidium bromide along with a 1-kb plus DNA ladder (Invitrogen).

### Indirect Immunofluorescence Assay

For indirect immunofluorescence assays, cells were grown on two well chamber slides from Nunc (Fisher scientific, Rochester, NY). The cells were fixed with 3.75% formaldehyde in PBS for 20 min on ice. After three additional washes with PBS, the plasma membrane was permeabilized with 0.1% Triton X-100; 10 mM Tris HCl at PH 7.5, 120 mM NaCl; 25 mM KCl; 2 mM EGTA; and 2 mM EDTA for 10 min at room temperature. Cells were incubated with 3% Bovine serum albumin (Sigma) in PBS before incubation with primary antibodies. Rabbit anti-ZO-1 antibody (61-7300 from Invitrogen) and rabbit anti-E-Cadherin (C212 from Santa Cruz Biotechnology) were used at a 1:400 dilution. Secondary Alexa 488 anti-rabbit (Molecular Probes, Inc., Eugene, OR) were used at a 1:400 dilution. Stained cells were mounted with Vectashield Mounting media containing DAPI (Vector Laboratories, Inc., Burlingame, CA). Stained and mounted cells were then processed with a Zeiss Axioplan epifluorescence microscope (Carl Zeiss, Thornwood, NY).

### Transfection of Ishikawa cells

To generate stably transfected cells, Ishikawa cells at passage number 25, were transfected with either 0.2 μg of CMV-neo empty vector or CMV-HER2 (CMV empty vector and CMV-HER2 were generously provided by the laboratory of Dr. Bjeldanes, UC Berkeley, CA, USA), using polyfact (Qiagen, CA) and following the manufacturer's suggested protocol. Cells were fed 24 h after transfection with DMEM, supplemented with 10% fetal calf serum, penicillin/streptomycin. The media was replaced with same media containing 0.7 mg/ml G418 (neomycin analog, Mediatech, Herndon, VA) to select for transfected cells. Selection media was replaced every 24 hours for a month and surviving cell populations were propagated in selection media. Experimental treatments were not performed in selection media.

### Subcellular Fractionation

The nuclear and nonnuclear subcellular fractions were harvested from cell extracts using the NE-PER Nuclear Cytoplasmic Extraction Reagents (Pierce, Rockford, IL) according to the manufacturer's instruction. The total protein was quantified using Bradford reagents (BioRad). Cell fractions were examined by Western blots as described above. Anti-lamin was used as a marker for nuclear fraction.

## Results

### Effects of Arecoline on expression of the ZO-1 tight junction protein and the HER2 tyrosine kinase receptor

**Figure 1:**
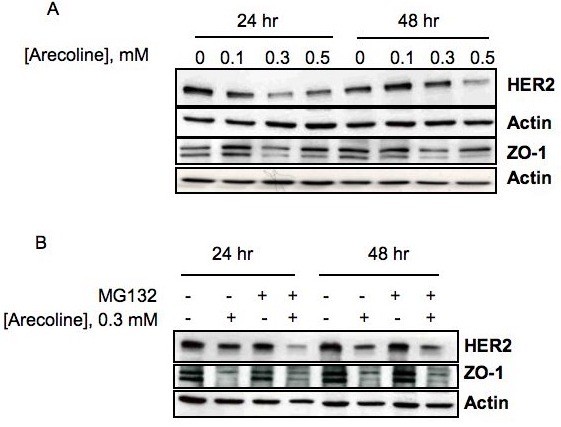
**Effects of arecoline on expression of ZO-1 and HER2 protein in Ishikawa cells**. (A) Subconfluent cultures of Ishikawa cells were treated with DMSO (vehicle control), 0.1 mM, 0.3 mM or 0.5 mM arecoline for 24 and 48 hrs, and total cell extracts were fractionated in SDS polyacrylamide gels. The arecoline regulation of ZO-1 and HER2 protein production was determined by western blot analysis and compared to the levels of actin protein; (B) To determine if the arecoline mediated downregulation of ZO-1 and HER2 protein was due to induced ubiquitination and 26 S proteasome mediated degradation, Ishikawa cells were treated with or without 0.3 mM arecoline for 48 hrs and in the presence or absence of MG132, an inhibitor of proteasome peptidase enzymatic activity. Total cell extracts were analyzed by Western blotting for ZO-1 and HER2 in comparison to actin.

Arecoline has been detected in saliva obtained during betel nut chewing in concentrations up to 140 μg/ml, corresponding to 0.9 mM. Arecoline in the millimolar concentration range is thought to participate in the initiation and/or progression of cellular changes during the long-term effects of betel nut chewing [25]. Therefore, to examine the potential effects of arecoline on expression of the ZO-1 tight junction protein and HER2 member of the EGF receptor gene family, cultured human Ishikawas endometrial cancer cells were treated with concentrations of arecoline ranging between 0.1 mM and 0.5 mM and the production of ZO-1 and HER2 protein determined by western blot analysis. The Ishikawa cells were treated for 24 and 48 hr with each arecoline concentration and compared to a DMSO vehicle treated control (0 mM arecoline). As shown in Figure 1A, arecoline treatment down-regulated production of both ZO-1 and HER2 protein that was observed within 24 hours of treatment at 0.3 mM arecoline. Under the conditions of this experiment, there were no observed changes in actin production, which also serves as a gel loading control. In most of our study, we employed 0.3 mM arecoline, which induces the maximum effect on ZO-1 and HER2 expression without causing apoptosis.

To determine if the arecoline-induced loss of HER2 and ZO-1 protein was due to ubiquitin-26 S proteasome mediated degradation, Ishikawa cells were treated with or without 0.3 mM arecoline for 24 hr and 48 hr in the presence or absence of MG132, an inhibitor of proteasome peptidase enzymatic activity. As shown in Figure 1B, western blot analysis indicated that the downregulation of both ZO-1 and HER2 protein strongly occurs in the presence of MG132, suggesting that the loss of both proteins are not due to proteasomal degradation.

### Arecoline Downregulates ZO-1 and HER 2 Transcript Levels in Ishikawa endometrial cancer cells

**Figure 2:**
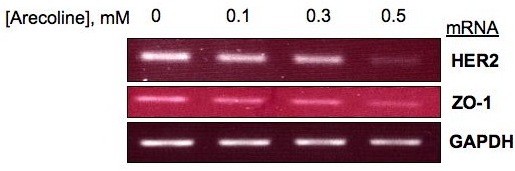
**Arecoline downregulates of ZO-1 and HER2 transcripts in Ishikawa cells**. Ishikawa cells were treated with the DMSO vehicle control, 0.1 mM, 0.3 mM or 0.5 mM arecoline for 48 hrs and total RNA was isolated and quantified by RT-PCR analysis. Oligonucleotides specific for ZO-1, HER2 or GAPDH were used to generate specific RT-PCR fragments that were fractionated in agarose gels. The transcript specific bands were visualized by ethidium bromide staining.

To uncover the cellular processes regulated by arecoline that leads to the down-regulation of ZO-1 and HER2 protein levels, Ishikawa cells were cultured in the presence of varying concentrations of arecoline for 48 hours, and the levels of HER2 and ZO-1 transcripts were compared with DMSO vehicle treated control cells. As shown in Figure 2, reverse transcription-PCR analysis revealed that arecoline treatment downregulates expression of HER2 and ZO-1 transcripts after 48 hours in a dose dependent manner. Maximum effects were observed after the cells were treated with 0.5 mM arecoline for 48 hr, although signaificant effects were observed in the presence of 0.3 mM arecoline. GAPDH transcript levels remained unchanged and were used as gel loading controls. The arecoline mediated loss of ZO-1 and HER2 transcripts accounts for the down-regulation of the corresponding protein levels.

### Effects of Arecoline on expression of Tight Junction and Adherens Junction proteins

**Figure 3:**
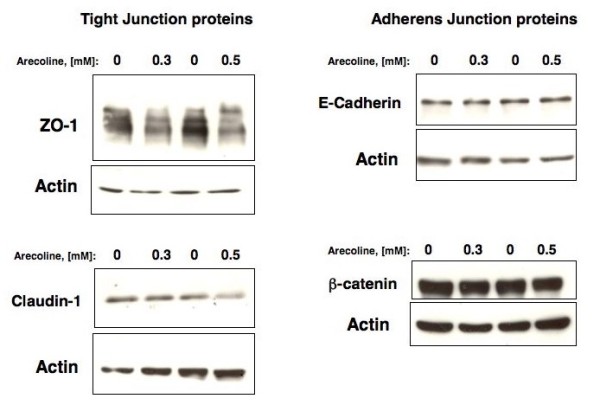
**Arecoline effects on expression of tight junction and adherens junction proteins in Ishikawa cells**. Ishikawa cells were treated with DMSO (vehicle control) or with either 0.3 mM or 0.5 mM arecoline for 48 hrs, and total cell extracts were fractionated in SDS polyacrylamide gels. The production of ZO-1, Claudin-1, E-cadherin, and beta-catenin protein was determined by western blot analysis and compared to the levels of actin protein.

Both tight junctions and adherens junction are comprised of distinct protein complexes [15], and therefore the potential effects of arecoline were assessed on expression of several tight junction and adherens junction proteins. Ishikawa cells were treated with or without 0.3 mM and 0.5 mM arecoline for 48 hours, and the level of the ZO-1 and Claudin-1 tight junction proteins and the E-cadherin and beta-catenin adherens junction proteins were analyzed by western blots. Actin proteins levels were used as a constitutive control protein for comparison to the apical junction proteins. As shown in Figure 3, under conditions in which arecoline strongly down-regulated ZO-1 protein levels, this alkaloid also down-regulated the Claudin-1 protein, which is also a component of the tight junction. Arecoline had no significant effects on the protein levels of either E-cadherin or beta-catenin, which are both critical components of adherens junctions. Thus, expression of tight junction proteins appears to be significantly more sensitive to the disruptive effects of arecoline compared to adherens junction proteins.

### Arecoline disruption of the localization of ZO-1 protein

**Figure 4:**
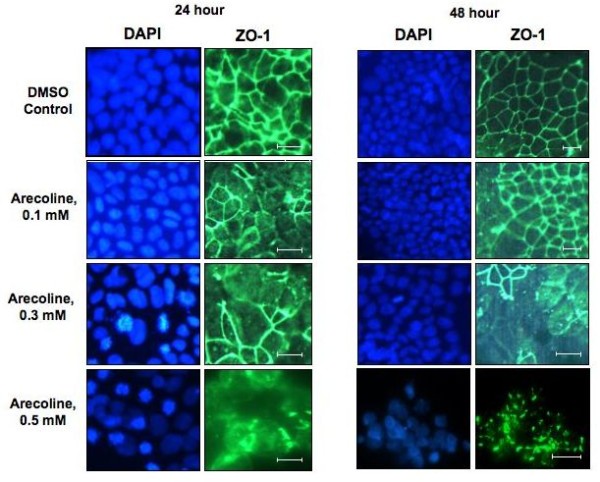
**Immunofluorescence analysis of the arecoline disruption of ZO-1 protein localization**. Ishikawa cells were treated with the DMSO vehicle control, 0.1 mM, 0.3 mM, or 0.5 mM arecoline for 24 and 48 hrs. Cells were fixed in 3.7% formaldehyde and stained for localization of ZO-1 by indirect immunofluorescence and for nuclear DNA by DAPI staining. Bars = 20 μm.

In the apical junction, ZO-1 characteristically forms a continuous band at the periphery of well-differentiated, confluent, polarized epithelial cells. The localization of ZO-1 changes dramatically according to the confluency of cells, with low confluent cells having an accumulation of nuclear ZO-1 localization and high confluent cells having ZO-1 locate at the plasma membrane. Nuclear localization of ZO-1 has also been detected in many cell types [26]. Indirect immunofluorescence was utilized to assess the potential effects of arecoline on ZO-1 localization in Ishikawa cells treated with various concentrations of arecoline for 24 hours and 48 hours. As shown in Figure 4, arecoline treatment disrupted the characteristically continuous bands of ZO-1 staining around the apices of Ishikawa cells. Treatment with 0.3 mM arecoline induced a near maximal effect on ZO-1 localization at both 24 hours and 48 hours of incubation. The overall ZO-1 staining pattern in arecoline treated cells was highly disorganized, which is indicative of a disruption of the apical junctional complex.

**Figure 5:**
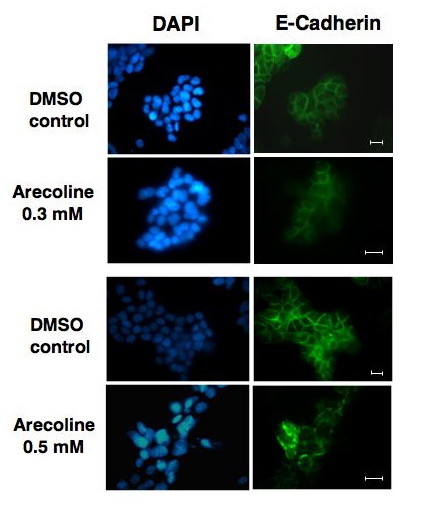
**Immunofluorescence analyiss of arecoline effects on the cellular staining pattern of E-cadherin**. Ishikawa cells were treated with the DMSO vehicle control or with either 0.3 mM, or 0.5 mM arecoline for 48 hrs. Cells were fixed in 3.7% formaldehyde and stained for localization of E-cadherin by indirect immunofluorescence and for nuclear DNA by DAPI staining. Bars = 20 μm.

The effect of arecoline on the ZO-1 cellular staining pattern was examined in the context of the cellular staining pattern of the adherens junction protein E-cadherin. Ishikawa cells were treated with or without 0.3 mM and 0.5 mM arecoline for 48 hours and the E-cadherin staining pattern analyzed by indirect immunofluorescence. As shown in figure 5, at the lower arecoline concentration of 0.3 mM, the overall E-cadherin staining pattern remained mostly intact and highly organized to the cell periphery. At this alkaloid concentration, the ZO-1 staining pattern was mostly disorganized (shown in Figure 4). At the higher arecoline concentration (0.5 mM), E-cadherin shows a generally similar degree of disorganization as that observed for ZO-1 (Figure 5 compared to Figure 4).

**Figure 6:**
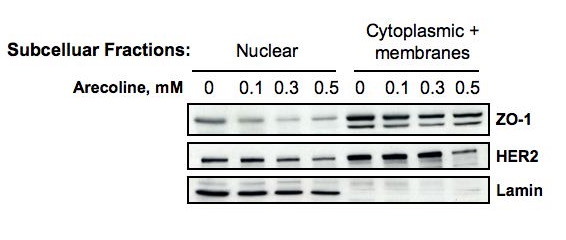
**Arecoline down regulates the level of ZO-1 and HER2 proteins in both the Cytoplasmic/membrane as well as nuclear fractions of Ishikawa cells**. The Ishikawa cells were treated with the DMSO vehicle control, 0.1 mM, 0.3 mM, or 0.5 mM arecoline for 48 hours. The nuclear and cytoplasmic/memebrane fraction was separated biochemically by differential centrifugation, and distribution of ZO-1 and HER2 was evaluated by western blot analysis in comparison to the lamin nuclear marker protein.

To further characterize the effects of arecoline on the subcellular distribution of ZO-1, the nuclear and cytoplasmic/membrane fractions were biochemically separated after treatment of Ishikawa cells for 48 hr with 0 mM (vehicle treated control), 0.1 mM, 0.3 mM or 0.5 mM arecoline. ZO-1 exists as two isoforms depending upon the presence or absence of an 80 amino acid N-terminal domain denoted as ZO-1 α^+ ^and ZO-1 α^- ^[15]. The proportion of each isoform is characteristic of particular cell types [15]. As shown in Figure 6, the nuclear fraction of Ishikawa cells was found to contain only ZO-1 α^+ ^whereas, the cytoplasmic/membrane fraction contains both ZO-1 α^+ ^and ZO-1 α^-^. Arecoline treatment down-regulated the expression of both isoforms from the nuclear as well as cytoplasmic/membrane subcellular fractions compared to the DMSO vehicle treated control cells (0 mM arecoline). HER2 can be imported into the nucleus of certain cell types through a nuclear localization signal mediated mechanism [27]. Our results also revealed that HER2 is localized in both the cytomplasmic/membrane and the nuclear fractions of the Ishikawa cells, and the treatment with arecoline down-regulates HER2 protein levels from both subcellular fractions (Figure 6).

### Expression of exogenous HER2 prevents the arecoline down-regulation of ZO-1 and overrides the disruption of ZO-1 localizalization in Ishikawa cells

**Figure 7:**
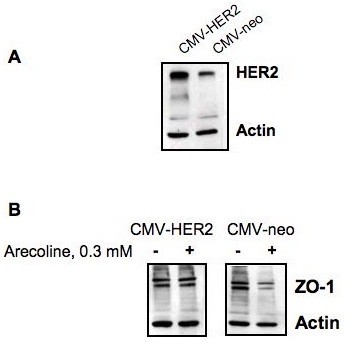
**Expression of exogenous HER2 prevents the arecolin down-regulation of ZO-1 expression in Ishikawa cells**. (A) Ishikawa cells were stably transfected with either the CMV-HER2 expression plasmid or the CMV-neo empty vector control plasmids and transfection competent cells selected in media containing G418. Western blotting shows that the in CMV-HER2 transfected cells, HER2 protein levels are over-expressed compared to cells transfected with the CMV-neo empty vector. (B) CMV-HER2 transfected and CMV-neo transfected cells were treated with or without 0.3 mM arecoline for 48 hrs and the level of ZO-1 protein was determined by western blotting. The level of actin protein was used as a gel loading control.

To functionally test the link between the arecoline down-regulated expression of ZO-1 and HER2, Ishikawa cells were transfected with the CMV-HER2 expression plasmid or with the control CMV-neo empty vector plasmids. The transfection competent cells were stably selected for 30 days in G418. Western blot analysis revealed that CMV-HER2 transfected cells expressed significantly higher levels of HER2 protein compared to the control transfected cells (Figure 7A). Both the CMV-HER2 transfected and the CMV-neo transfected Ishikawa cells were treated with 0.3 mM arecoline for 48 hours, and the production of ZO-1 protein was examine by western blot analysis. As shown in figure 7B, expression of exogenous HER2 ablated the arecoline down-regulation of ZO-1 protein, which demonstrates a strong functional connection between the level of HER2 protein and ability of arecoline to attenuate ZO-1 expression.

**Figure 8:**
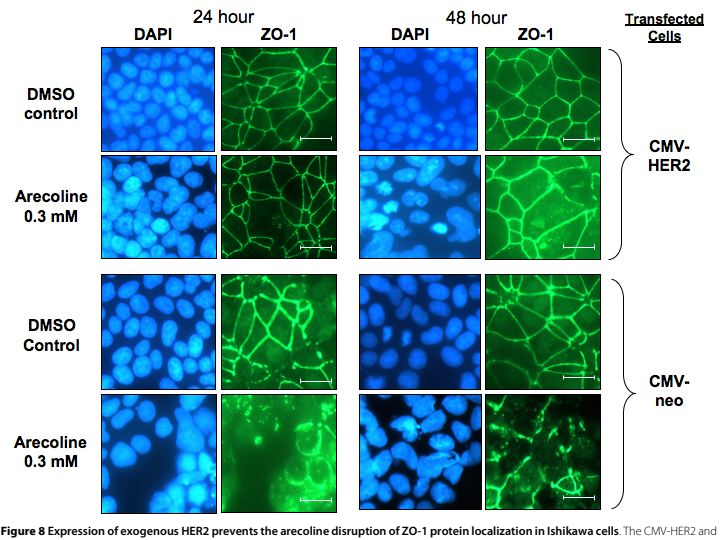
**Expression of exogenous HER2 prevents the arecoline disruption of ZO-1 protein localization in Ishikawa cells**. The CMV-HER2 and CMV-neo empty vector transfected Ishikawa cells were treated with the DMSO vehicle control or with 0.3 mM Arecoline for 24 hours and 48 hours. Cells were fixed in 3.5% formaldehyde and stained for the localization of ZO-1 by indirect immunofluorescence or for nuclear DNA by DAPI staining. Bars = 20 μm.

Indirect immunofluorescence was employed to examine the potential effects of exogenous HER2 expression on the arecoline disruption of ZO-1 localization. As shown in Figure 8, in CMV-HER2 transfected Ishikawa cells, expression of exogenous HER2 completely prevented the arecoline-mediated disruption of ZO-1 localization to the cell periphery. The ZO-1 staining pattern in CMV-HER2 cells treated with arecoline for 24 hours or 48 hours was virtually identical to DMSO vehicle treated control cells (Figure 8, upper panels). As expected, in the CMV-neo control transfected cells, arecoline treatment induced a significant disruption of ZO-1 localization (Figure 8, lower panels), with the staining pattern indicative of a disorganized junctional complex. It is important to point that the ZO-1 staining pattern in arecoline treated and untreated CMV-neo transfected cells is essentially the same as that observed in untransfected cells (Figure 4) showing that the transfection per se had no unusual effect on the cell phenotype.

### Dexamethasone treatment overrides the arecoline disruption of ZO-1 localization in Ishikawa cells

**Figure 9:**
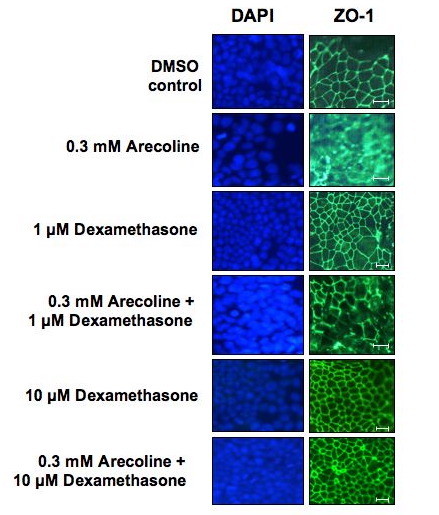
**Treatment with dexamethasone overrides the arecoline disruption of ZO-1 protein localization in Ishikawa cells**. Ishikawa cells were incubated with or without 1 μM or 10 μM of dexamethasone in the presence or absence of 0.3 mM Arecoline. Cells were fixed in 3.5% formaldehyde and stained for the localization of ZO-1 by indirect immunofluorescence or for nuclear DNA by DAPI staining. Bars = 20 μm.

It has been previously shown that treatment with the synthetic glucocorticoid dexamethasone strongly stimulates expression of HER2 in Ishikawa endometrial cancer cells and in human epithelial ovarian carcinoma cell lines [24]. Our previous study demonstrated that dexamethasone induces tight junctional complex formation in the rat Con8 mammary epithelial tumor cell line [28]. Together, these observations suggest that dexamethasone treatment may provide a hormonal tool to functionally assess the arecoline effects on the dynamics of ZO-1 localization. Ishikawa cells were treated with or without 1 μM dexamethasone or 10 μM dexamethasone and apical junction localization of ZO-1 was visualized using indirect immunofluorescence. As shown in Figure 9, ZO-1 staining revealed that in dexamethasone treated cells, the apical junction complex was somewhat more organized compared to the DMSO treated cells. In presence of 1 μM dexamethasone, the disruptive effects of 0.3 mM arecoline on ZO-1 localization were partially restored (Figure 9). At 10 μM dexamethasone, the disruptive effects of 0.3 mM arecoline was completely ablated as the ZO-1 staining pattern in cells treated with dexamethasone and arecoline was quite similar to that observed in the DMSO vehicle treated control cells (Figure 9, top versus bottom panels).

## Discussion

We have established that arecoline has profound effects on plasma membrane associated signaling proteins in the human endometrial Ishikawa cell line. Arecoline was shown to coordinately down regulate the expression and disrupt localization of the ZO-1 tight junction component of the apical junction complex as well as decrease expression of the HER2 member of the epidermal growth factor receptor gene family. Our studies have uncovered a functional link between the arecoline down regulation of ZO-1 and HER2 because expression of exogenous HER2 completely prevents the ability of arecoline to disrupt ZO-1 expression and localization to the cell periphery. Furthermore, treatment with dexamethasone, a synthetic glucocorticoid that has been shown to upregulate HER2 expression in Ishikawa endometrial cancer cells [24], also overrides the disruptive effects of arecoline on ZO-1 localization. A functional connection between HER2 levels and the control of ZO-1 localization or expression has not been previously observed in human cancer cells.

HER2 plays an important role in the regulation of cell growth, differentiation and survival through its heterodimerization with other members of the EGF receptor gene family [29]. A variety of cell and tissue types expresses HER2 [29], and a number of human cancers frequently over-express HER2 due to gene amplification including many reproductive cancers [21,30-33] as well as lung, gastric and oral cancers [34-39]. Patients with HER2-overexpressing breast or ovarian cancer have significantly shorter overall survival rate and time of relapse relative to patients with tumors without HER2 overexpression [21,30,31]. Because of HER2 overexpression in many cancers, its accessible location on the cell surface and its role in carcinogenesis HER2 has been under intensive scrutiny as a therapeutic target. HER2 is expressed at low levels in normal tissue compared to cancer cells [40], which suggests the existence of a suitable therapeutic window to minimize damage to normal cells but still be able to target HER2-positive cancers by inhibiting either HER2 protein function or expression [41].

Studies examining ZO-1 protein stability have uncovered a range of ZO-1 protein half lives (ranging between 5 and 20 hours) that can differ depending on the cell type and cell cultured conditions such as cell confluency [42,43]. Although in many systems, regulated changes in the stability of ZO-1 protein can potentially play a role in its cellular regulation, we have shown that in Ishikawa endometrial cancer cells, the loss of ZO-1 protein is accounted for an a corresponding loss in ZO-1 transcript levels. We have also determined that arecoline concurrently reduces HER2 protein and transcript expression along with that of ZO-1 expression, and that ectopic expression of HER2 reverses the arecoline down regulation of ZO-1. We are currently attempting to establish the precise mechanism by which the arecoline-mediated loss of HER2 levels leads to these effects on ZO-1 utilization. In this regard, is thought that over-expression of HER2 in human cancer cells due to amplification enhances the preferential binding of the low-affinity arm of ligands to HER2 resulting in increased intracellular signaling [44] that could ultimately lead to the control of ZO-1 and potential regulation of ZO-1 mediated cell-cell interactions. Interestingly, a transcriptional factor that binds to the SH3 domain of ZO-1 (ZONAB, ZO-1-associated nucleic acid binding protein) was shown in MDCK cells to functionally interact with the nuclear form of ZO-1 to modulate expression of HER2 in a cell density dependent manner [45]. This study, in combination with our results, suggests that the expression and cellular use of ZO-1 and HER2 may be linked thorough a mutual feedback system in certain human cancer cells. Because dexamethasone, a synthetic glucocorticoid, regulates the transcription of glucocorticoid receptor target genes and overrides the effects of arecoline on ZO-1 localization, it is tempting to speculate that this steroid hormone alters the transcriptional dynamics of HER2 in this system and thereby stabilizes ZO-1 expression and localization.

## Conclusion

Arecoline induced cellular changes in the oral cavity in areca nut chewers leading to oral precancerous lesions may be due to disrupted expression and junctional localization of the ZO-1 tight junctional protein. Furthermore, we have established that the ability of arecoline to control ZO-1 in human Ishikawa cancer cells requires the coordinate down regulation of the HER2 member of the EGF receptor gene family. This observation represents a previously unknown functional connection between HER2 expression and the cellular accessibility of ZO-1. Thus, the physiological control of HER2 expression in human tissues may play a direct role in the susceptibility of humans to the carcinogenic effects of arecoline.

## Authors' contributions

SG and GLF jointly designed the experiments and wrote the paper. SG completed all of the ZO-1 protein experiments. SNS helped in designing the experiments and KMP conducted the RT-PCR study and the western blots and indirect immunofluorescence of the adherens junction proteins. All authors read and approved the final manuscript.
